# Skewed Distribution of IL-7 Receptor-α-Expressing Effector Memory CD8^+^ T Cells with Distinct Functional Characteristics in Oral Squamous Cell Carcinoma

**DOI:** 10.1371/journal.pone.0085521

**Published:** 2014-01-23

**Authors:** Jang-Jaer Lee, Chiou-Yueh Yeh, Chiau-Jing Jung, Ching-Wen Chen, Mao-Kuang Du, Hui-Ming Yu, Chia-Ju Yang, Hui-yi Lin, Andy Sun, Jenq-Yuh Ko, Shih Jung Cheng, Yen-Liang Chang, Jean-San Chia

**Affiliations:** 1 Department of Oral Maxillofacial Surgery, National Taiwan University Hospital, Taipei, Taiwan, ROC; 2 Graduate Institute of Immunology, College of Medicine, National Taiwan University, Taipei, Taiwan, ROC; 3 Genomic Research Center, Academic Sinica, Taipei, Taiwan, ROC; 4 Graduate Institute of Microbiology, College of Medicine, National Taiwan University, Taipei, Taiwan, ROC; 5 Department of Otolaryngology, National Taiwan University Hospital, Taipei, Taiwan, ROC; 6 Fu Jen Catholic University, School of Medicine, and Department of Otolaryngology, Cathay General Hospital, Taipei, Taiwan, ROC; Purdue University, United States of America

## Abstract

CD8^+^ T cells play important roles in anti-tumor immunity but distribution profile or functional characteristics of effector memory subsets during tumor progression are unclear. We found that, in oral squamous carcinoma patients, circulating CD8^+^ T cell pools skewed toward effector memory subsets with the distribution frequency of CCR7^−^CD45RA^−^CD8^+^ T cells and CCR7^−^ CD45RA^+^CD8^+^ T cells negatively correlated with each other. A significantly higher frequency of CD127^lo^ CCR7^−^CD45RA^−^CD8^+^ T cells or CCR7^−^CD45RA^+^CD8^+^ T cells among total CD8^+^ T cells was found in peripheral blood or tumor infiltrating lymphocytes, but not in regional lymph nodes. The CD127^hi^ CCR7^−^CD45RA^−^CD8^+^ T cells or CCR7^−^CD45RA^+^CD8^+^ T cells maintained significantly higher IFN-γ, IL-2 productivity and ex vivo proliferative capacity, while the CD127^lo^ CCR7^−^CD45RA^−^CD8^+^ T cells or CCR7^−^CD45RA^+^CD8^+^ T cells exhibited higher granzyme B productivity and susceptibility to activation induced cell death. A higher ratio of CCR7^−^CD45RA^+^CD8^+^ T cells to CCR7^−^CD45RA^−^CD8^+^ T cells was associated with advanced cancer staging and poor differentiation of tumor cells. Therefore, the CD127^lo^ CCR7^−^CD45RA^−^CD8^+^ T cells and CCR7^−^CD45RA^+^CD8^+^ T cells are functionally similar CD8^+^ T cell subsets which exhibit late differentiated effector phenotypes and the shift of peripheral CD8^+^ effector memory balance toward CCR7^−^CD45RA^+^CD8^+^ T cells is associated with OSCC progression.

## Introduction

CD8^+^ T cells play important roles in mediating anti-tumor immunity and adoptive transfer-based immunotherapy may achieve regression of tumors [1]. Oral cancer, which is primarily squamous cell carcinoma (OSCC), is the fifth most common cancer world-wide [2]. Compared to conventional treatment for oral or head and neck cancers, adoptive transfer-based immunotherapy is a relatively specific approach directed to tumor cells through the activated effectors such as CD8^+^ T cells, in an antigen-dependent manner [3]–[4]. In patients with oral or head and neck squamous cell carcinoma, wild-type p53-specific cytotoxic CD8^+^ T cells play a direct role in the elimination of tumor cells expressing the p53_264–272_ epitope and in immunoselection of epitope-lost tumor cells carrying mutated-p53 [5]. Interestingly, cytotoxic CD8^+^ T cells in metastatic lymph nodes, but not in tumor infiltrating lymphocytes, are associated with favorable outcome in patients with OSCC [6], implicating that CD8^+^ T cells could mediate systemic protective immune response despite of the immunoselection or immunosuppression occurred locally in OSCC microenvironment [7]–[8].

Human peripheral CD8^+^ T cells are heterogeneous populations and could be identified by their surface expression of glycoproteins (e.g. CCR7, CD45RA), or costimulatory molecules (e.g. CD27, CD28) [9]–[11]. Naive CD8^+^ T cells express high molecular weight isoforms of leukocyte common antigen CD45RA, CD28 and CCR7, a lymph-node-homing chemokine receptor. Human memory CD8^+^ T cells express the low molecular weight isoform of the common leukocyte antigen CD45RO and can be classified into CCR7^+^ “central memory” cells and CCR7^−^“effector memory” cells [9]. However, CD45RA, originally considered to be marker for naive CD8^+^ T cells, can also found in human memory CD8^+^ T cells, which have been termed “effector memory RA” (T_EMRA_) or “revertant memory” cells because of their re-expression of CD45RA and effector memory-like phenotypes [12]. Interestingly, the T_EMRA_ may resume proliferative responses after receiving the appropriate costimulatory signals [13]. A most recent report indicated that the low frequency of circulating CD8^+^ CCR7^+^ T cells is a significant risk factor for tumor recurrence in patients with head and neck cancer [14], suggesting that skewed distribution of functionally distinct CD8^+^ T-cell subset may occur during cancer progression.

Circulating CD8^+^ T cells in oral or head and neck cancer patients have been well-characterized for their susceptibility to apoptosis and the responsible Fas/FasL or TRAIL/TRAILR signaling pathway [15]–[16]. However, the distribution profiles or functional characteristics of the specific CD8^+^ T-cell subsets in either tumor infiltrating lymphocytes or systemic circulation of cancer patients are still unclear. Moreover, the identification or isolation of specific effector memory subsets exhibiting ex vivo proliferative capacity and resistant to activation induced cell death is important clinically for conducing adoptive-transfer based cancer immunotherapy [17]. IL-7 signaling occurs through the IL-7 receptor (IL-7R) complex, which is composed of the IL-7Rα chain (CD127) and the common cytokine receptor γ-chain (γc or CD132) [18]–[19]. IL-7 signaling promotes human CD8^+^ T cell generation and cytolytic reactivity [20]–[21]. Importantly, recombinant IL-7 administration achieved an increase in the peripheral CD4^+^ or CD8^+^ T cell numbers and could significantly improves lymphocyte functionality, including proliferation and IFN-γ production [22]. In this study, we demonstrated that IL-7 receptor expression on CD8^+^ T cells may differentiate the CD45RA^+/−^ CCR7^−^ effector memory CD8^+^ T-cell subsets that exhibit increased distribution frequency, distinct functional characteristics and association with clinicopathological status for tumor progression in OSCC patients.

## Materials and Methods

### Patients and healthy donors

In total, 59 patients with OSCC, who were seen between July 2011 and June 2013 at the Department of Oral Maxillary Facial Surgery or Otolaryngology Clinic at the National Taiwan University Hospital (NTUH) were enrolled into the study. Eighteen normal healthy donors (HD) were recruited among the laboratory personnel and other volunteers. Detailed information of all donors is summarized in Table 1. The Institutional Review Board of NTUH has approved the protocol for collection of patient blood samples or tumor specimens and written informed consent was obtained from each individual participating in this study. All the patients and control subjects were not receiving any medications and had not received any therapy before entering the study.

**Table 1:** Correlations of distinct subsets or ratio of CD8^+^ T cells with tumor progression.

	T		N	Stage	Differentiation	ECS	LVI	PNI
CCR7^+^CD45RA^+^CD8^+^ T cell	0.055 (0.689)	−0.023 (0.874)		0.02 (0.885)	0.024 (0.869)	−0.059 (0.669)	0.208 (0.127)	0.076 (0.579)
CCR7^+^CD45RA^−^CD8^+^ T cell	−0.031 (0.823)	0.097 (0.505)		−0.019 (0.892)	0.067 (0.629)	0.047 (0.731)	0.017 (0.901)	0.001 (0.992)
CCR7^−^CD45RA^−^CD8^+^ T cell	−0.022 (0.871)	−0.2 (0.164)		−0.297 (0.026*)	−0.298 (0.027*)	−0.057 (0.678)	−0.148 (0.282)	−0.22 (0.107)
CCR7^−^CD45RA^+^CD8^+^ T cell	0.202 (0.135)	0.206 (0.151)		0.276 (0.04*)	0.183 (0.18)	0.105 (0.446)	−0.036 (0.796)	0.132 (0.336)
CD127^lo^CCR7^−^ CD45RA^−^CD8^+^ T cell	0.087 (0.254)	−0.205 (0.153)		−0.026 (0.846)	−0.15 (0.274)	−0.175 (0.201)	−0.133 (0.334)	−0.123 (0.371)
CD127^lo^CCR7^−^ CD45RA^+^CD8^+^ T cell	−0.068 (0.621)	−0.247 (0.084)		−0.186 (0.17)	−0.096 (0.488)	−0.071 (0.605)	−0.155 (0.259)	−0.17 (0.216)
CCR7^−^CD45RA^+^CD8^+^ T cell/CCR7^−^ CD45RA^−^CD8^+^ T cell ratio	0.23 (0.089)	0.244 (0.087)		0.279 (0.037*)	0.338 (0.012*)	0.074 (0.593)	−0.021 (0.882)	0.081 (0.557)

T, tumor size; N, positive nodular metastasis; ECS, extra-capsular spread; LVI, lymphovascular invasion; and PNI, peri-neural invasion.

(*P* value*) Pearson and Spearman correlation tests *P* value <0.05.

### PBMC collection, peripheral CD8^+^ T cell isolation

Venous blood obtained from patients or healthy donors (20–30 ml) was delivered to the laboratory within 30 min of venipuncture. Plasma was collected by centrifugation for IL-7 analysis and the peripheral mononuclear cells (PBMCs) were prepared by Ficoll-Paque Plus^TM^ gradient (GE healthcare) and CD8^+^ T cells were enriched by negative selection (RosetteSep, Stemcell Technologies Inc.). IL-7 was determined by sandwich ELISA kit (Peprotech).

### Isolation of lymphocytes from tumor and lymphoid node

Tumor infiltrated lymphocytes were enriched by Percoll discontinuous gradients (3 different concentrations; 20%, 55%, and 100%) after pressing tissues gently through a 40 μm sieve. Single-cell suspensions from lymphoid node or tumor infiltrated lymphocytes and flow cytometric analysis were performed as we have described previously [23].

### Solid-phase peptide synthesis and antigen specific response

Thirteen putative HLA-A*0201 binding peptides derived from alpha-enolase including amino acid 57–72, 76–84, 92–100, 110–137, 139–152, 162–176, 187–195, 216–224, 307–323, 339–347, 349–357, and 403–417 were predicted by ProPred I [24]. These peptides were synthesized, via 4- (2,4-dimethoxyphenyl-Fmoc-amino-methyl) phenoxyl resin (0.281 g, 0.89 mEq/g) with Fmoc-amino acid derivatives using an automatic peptide synthesizer (Applied Biosystem Model 433A,USA). After completion of synthesis, the peptide on resins were incubated with a cleavage mixture containing 0.75 g crystalline phenol, 0.25 mL 1,2-ethandithol, acetic acid, for 90 min at room temperature, and the solvent was completely evaporated. The dry resin was then washed five times with 20 mL of cold ether. Synthetic peptide was then extracted by washing five times with 20 mL of 5% acetic acid. All extracts were lyophilized to yield a crude peptide. The peptide was purified by HPLC using a C18 column (10-μm particle size, 250×10 mm) with a gradient (5% to 95% buffer B in 30 min) using buffer A (0.1% TFA in water) and buffer B (0.1% TFA in acetonitrile) at a flow rate of 2.5 mL/min and monitored by absorbance at 214 nm. Mass spectra were determined using a Bruker Ultraflex II TOF/TOF200 MALDI-TOF System. The HLA-A*0201 binding motif 264-272 of p53 (IBA, Life Science) was also included in antigen specific response [5]. PBMC were cultured with enolase peptide mixture or p53 specific peptide for five days, subsequently the interferon-gamma production in specific CD8^+^ subsets were performed by surface and intra-cellular staining as described [25]. CCR7^−^CD45RA^+^CD8^+^ T cell and CCR7^−^CD45RA^−^CD8^+^ T cell were selected for IFN-γ production. The data was presented as the frequency (%) of IFN-γ producing cells normalized by subtraction of the % of isotype control.

### Surface and intracellular staining

The following fluorochrome-conjugated monoclonal antibodies (mAbs) were used for flow cytometric analyses: FITC-conjugates of anti-CD45RA (clone HI100, BD pharmingen); PE-conjugate of anti-CD57 (clone HCD57, Biolegend), anti-PD-1 (clone EH12.2H7, Biolegend), anti-CD28 (clone CD28.2, Biolegend); PerCp-conjugate of CD8 (clone SK1, BD pharmingen); APC-conjugate of CCR7 (clone 3D12, eBioscience); PE-Cy7-conjugate of CD127 (clone eBioRDR5, eBioscience); APC-Cy7-conjugate of CD3 (clone HIT3a, Biolegend). PBMC (at least 0.2×10^6^ cells) were stained with fluorochrome-labeled mAbs for 30 min at 4°C in 100 μl staining buffer (PBS+ 4% Hi-FBS). Appropriate isotype antibody controls were used for each sample. Cells were washed and examined by flow cytometry.

Intracellular staining for interferon-γ and Granzyme B was performed by stimulating PBMC with 10 ng/mL PMA (phorbol 12-myristate 13-acetate), 1 μg/ml ionomycin, and 2 μM monensin for 4 hrs. Briefly, cells were fixed and permeabilized with 1 ml fixation/permeabilization buffer (eBioscience). After extensive washing, cells were stained with anti-CD3-APC Cy7, anti-CD8-PerCp, anti-IFN-γ-FITC (clone 4S.B3, eBioscience), and anti-Granzyme B-PE (clone GB11, eBioscience) for 30 min at 4°C. Cells were further washed twice with permeabilization buffer and resuspended in staining buffer, and then immediately analyzed by flow cytometry. Appropriate isotype controls were included for each sample.

### Cell sorting

PBMCs or CD8^+^ T cells were stained for fluorescence-activated cell sorting with mAbs specific to CD45RA-FITC, CD127-PE (clone eBioRDR5, eBioscience), CCR7-APC (clone 3D12, eBioscience), and CD3-PE Cy7 (clone UCHT1, Beckman) as described previously [23] and were sorted with a FACSAria (BD Biosciences, San Jose, CA) through a service provided by the Cell Sorting Core Facility (National Taiwan University College of Medicine, Taipei, Taiwan). The acquisition and analysis gates were restricted to the lymphocyte gate as determined by characteristic forward and side scatter properties of lymphocytes. Forward and side scatter were set to a linear scale. For analysis, 1×10^5^ lymphocytes were acquired. Two sorting strategies to obtain CD8^+^ T cell subsets were used: CCR7^+^CD45RA^+^CD8^+^T cell, CCR7^−^CD45RA^+^CD8^+^ T cell, CCR7^−^CD45RA^−^CD8^+^ T cell, CCR7^+^CD45RA^−^CD8^+^ T cell or CD127^hi^CCR7^−^CD45RA^−^CD8^+^ T cell, CD127^lo^CCR7^−^CD45RA^−^CD8^+^ T cell, CD127^hi^CCR7^−^CD45RA^+^CD8^+^ T cell, CD127^lo^CCR7^−^CD45RA^+^CD8^+^ T cell, respectively.

### Cell proliferation

CFSE (Molecular Probes) labeling was performed as described previously [23]. Briefly, sorted CD8 T-cell subsets were labeled with CFSE and cultured with anti-CD3/anti-CD28 coupled Dynabeads (bead to cell ratio is 1∶2) for 5 days. Propidium iodide (PI) was used to identify dead cells, and analysis was done by flow cytometry.

### RNA preparation and quantitative real-time PCR

Sorted CD8^+^ T cell subsets were stimulated with anti-CD3/anti-CD28 coupled Dynabeads for 6 hrs. Total RNA was extracted from stimulated CD8^+^ T cells using the TurboCapture^TM^ mRNA kit (Qiagen) and reverse-transcribed with oligo(dT)_18_ primer using M-MLV Reverse Transcriptase (Promega). The expression of IFN-γ and IL-2 was monitored by real-time PCR using the KAPA SYBR FAST qPCR kit (KAPA Biosystems) using a 7500 Real-Time PCR System (Applied Biosystems). IFN-γ primers (forward: 5′-TGC AAT CTG AGC CAG TGC TT-3′; reverse: 3′-CAG GGT CAC CTG ACA CAT TCA A-5′) and IL-2 (forward: 5′-AAC TCC TGT CTT GCA TTG CAC TA-3′; reverse: 3′-TTG CTG ATT AAG TCC CTG GGT C-5′) were utilized. Target gene transcription levels were measured and related to *GAPDH* expression. The culture supernatants of different CD8^+^ T cell subsets were collected to determine IFN-γ and IL-2 production using FlowCytomix (eBioscience).

### Statistical analysis

Data were presented as mean (± SEM) and analyzed with the Student's t-test or The Mann-Whitney U test to compare the mean levels of cytokine production or mRNA expression after the particular treatments using Graphpad Prism 5 software (GraphPad Software Inc., San Diego CA). Differences with the p value <0.05 were considered statistically significant. By using the Pearson and Spearman correlation tests, statistical analysis was performed to determine the strength of the associations of distinct CD8+ subsets with clinicopathological parameters for tumor progression. Differences with the p value <0.05 were considered statistically significant.

## Results

### Skewed distribution of memory CD8^+^ T cell pool in OSCC patients

**Figure 1:**
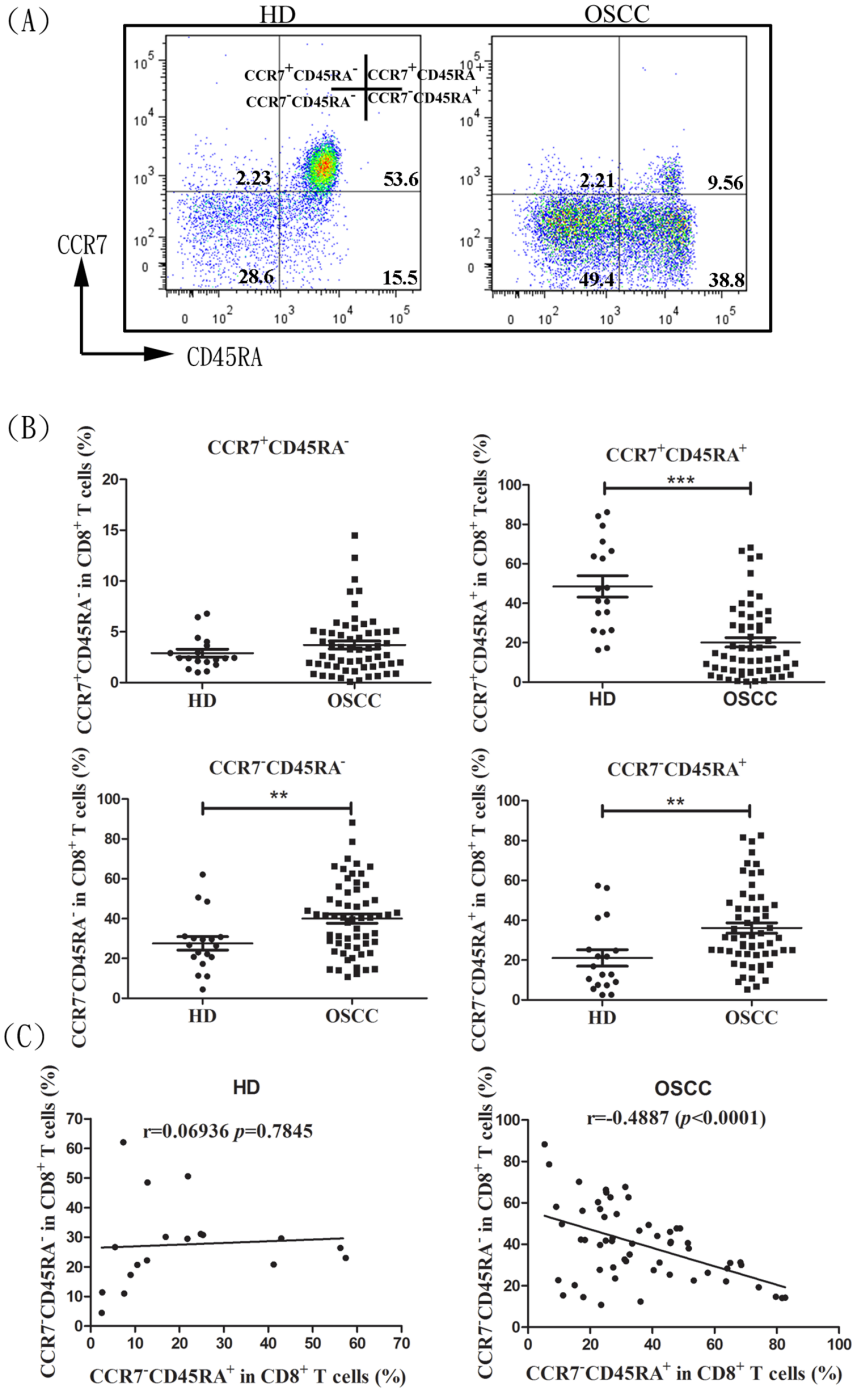
Distribution frequency and correlation of the effector memory CD8^+^ T cells in healthy donor and OSCC patients. (A) Peripheral CD8^+^ T cell can be separated into 4 subsets; CCR7^+^CD45RA^+^CD8^+^ T cell, CCR7^+^CD45RA^−^CD8^+^ T cell, CCR7^−^CD45RA^−^CD8^+^ T cell, and CCR7^−^CD45RA^+^CD8^+^ T cell based on the differential surface expression of CD45RA and CCR7 markers in healthy donor (left) and OSCC patient (right). (B) Distribution frequency (%) of different CD8^+^ T cell subsets within the total CD8^+^ T cells in the circulation of healthy donor (HD) or OSCC patients. (C) Correlation between the CCR7^−^CD45RA^−^CD8^+^ T cell and CCR7^−^CD45RA^+^CD8^+^ T cell subsets. B and C, the data are derived from 59 patients and 18 healthy donor. Significant difference was compared between each group using ***Mann-Whitney U*** test (*p<0.05, and ***p<0.001).

Human CD8^+^ T cells could be subdivided into four subsets: naive CCR7^+^CD45RA^+^; two memory subsets, central memory CCR7^+^CD45RA^−^ T cell or effector memory CCR7^−^CD45RA^−^T cell, and a fourth effector memory RA CCR7^−^CD45RA^+^T cell subset as previously described [9]–[10] (Fig. 1A). Based on the surface staining of CD45RA and CCR7, we could identify in peripheral blood from healthy donors (n = 18) similar subsets of CD8^+^ T cells within the CCR7^+^CD45RA^+^ population (48.53±5.4), followed by CCR7^−^CD45RA^−^CD8^+^ T cell (27.57±3.390%), CCR7^−^ CD45RA^+^CD8^+^ T cell (21.06±4.11%) and CCR7^+^CD45RA^−^CD8^+^ T cell (2.913±0.3839%) subsets (Fig. 1B). Interestingly, the dominance of the naive subset in circulating CD8^+^ T cell pool was skewed significantly toward CCR7^−^CD45RA^−^CD8^+^ T cell or CCR7^−^CD45RA^+^CD8^+^ T cell in OSCC patients (n = 59; Fig. 1B). OSCC patients exhibited higher CCR7^−^CD45RA^−^CD8^+^ T cell and CCR7^−^CD45RA^+^CD8^+^ T cell ratios, 40.06±2.35% and 36.10±2.58%, respectively, and compared to healthy controls. A decrease in the distribution frequency of the naive CD8^+^ subset was found in OSCC patients (20.14±2.38%). Interestingly, a negative correlation was found in the distribution percentage between CCR7^−^CD45RA^−^CD8^+^ T cell and CCR7^−^CD45RA^+^CD8^+^ T cell subsets in OSCC patients (Fig. 1C), suggesting that dynamic changes in circulating CCR7^−^CD45RA^+^CD8^+^ T cell and CCR7^−^CD45RA^−^CD8^+^ T cell occurred during cancer progression. These results indicated that peripheral CD8^+^ T cells skewed toward effector memory phenotypes during OSCC progression.

### Increased CD127-expressing proportions in skewed memory subsets

**Figure 2:**
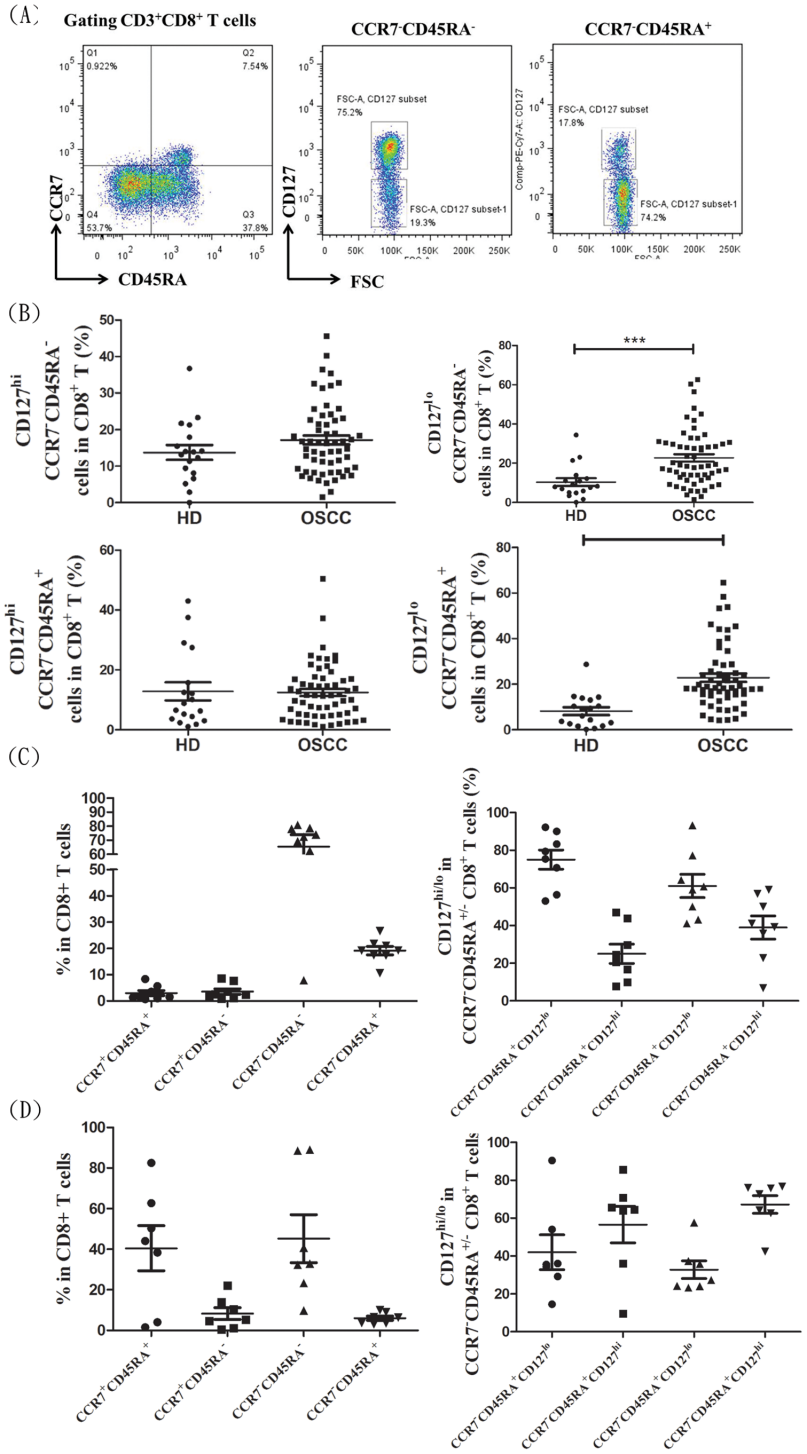
Distribution frequency of CD127^hi^ and CD127^lo^ CCR7^−^CD45RA^−^CD8^+^ T cell and CCR7^−^CD45RA^+^CD8^+^ T cell subsets in peripheral blood, tumor infiltrated lymphocytes and regional lymphoid nodes. (A) Differential expression of CD127 in CCR7^−^CD45RA^−^CD8^+^ T cell and CCR7^−^CD45RA^+^CD8^+^ T cell subsets and gating strategy for conducting cell sorting. The distribution frequency (%) of CD127^hi^ or CD127^lo^ CCR7^−^CD45RA^−^CD8^+^ T cell and CCR7^−^CD45RA^+^CD8^+^ T cell subsets in total peripheral CD8^+^ T cells from healthy donors; HD, n = 18, OSCC, n = 59 (B), tumor infiltrating lymphocytes; n = 8 (C) or regional lymph nodes; n = 7 (D). Data are plotted as the mean ± SD from HD and OSCC patients. Significant differences compared with each group using ***Mann-Whitney U*** **test** are indicated by asterisks (***p<0.001).

The human CCR7^−^CD45RA^−^CD8^+^ T cell or CCR7^−^CD45RA^+^CD8^+^ T cell exhibit altered expression of the CD127 (IL-7Rα chain) and elderly persons had decreased expression of CD127 in CCR7^+^CD45RA^+^ CD8^+^ or CCR7^−^CD45RA^+^CD8^+^ T subsets [26]. CD127 was consistently expressed on the majority of CCR7^+^CD45RA^+^CD8^+^ T cell and CCR7^+^CD45RA^−^CD8^+^ T cell populations in patients or healthy donors (90.26±13.1% and 82±16.6%, respectively). The average expression level (based on MFI) of CD127 showed no difference between different subsets of CD8^+^ T cells isolated from OSCC patients or healthy donors (data not shown). The expression levels of CD127 on either CCR7^−^CD45RA^−^CD8^+^ T cell or CCR7^−^CD45RA^+^CD8^+^ T cell were variable and could be divided into CD127^high (hi)^ or CD127^low (lo)^ populations (Fig. 2A). In OSCC patients, the proportions of CD127^ lo^ CCR7^−^CD45RA^−^CD8^+^ T cell or CD127^ lo^ CCR7^−^CD45RA^+^CD8^+^ T cell in total CD8^+^ pool exhibited a significant increase compared to those found in healthy donors (Fig. 2B). The distribution frequency of CD127^ lo^ subsets of CD8^+^ T cells is positively correlated with the increase of age (Figure S1 in File S1). But the distribution frequencies of these CD127^ lo^ subsets among total CD8^+^ T cells in OSCC patients are persistently higher than those found in the age-matched healthy donors (age below 45; Figure S2 in File S1).Therefore, such difference in CD127^ lo^ subsets seen between the groups of OSCC and healthy is regulated not only by immune status but also age.

Notably, the CCR7^−^CD45RA^−^CD8^+^ T cell and CCR7^−^CD45RA^+^CD8^+^ T cell are also found to be the major CD8^+^ subsets in tumor infiltrating lymphocytes with a significantly higher amount of CCR7^−^CD45RA^−^CD8^+^ T cell than CCR7^−^CD45RA^+^CD8^+^ T cell (Fig. 2C). Interestingly, similar to the circulating CD8^+^ pool, the CD127^ lo^ CCR7^−^CD45RA^−^CD8^+^ T cell or CCR7^−^CD45RA^+^CD8^+^ T cell in tumor infiltrates outnumbered by their CD127^ hi^ counterparts. In contrast, the CCR7^−^CD45RA^−^CD8^+^ T cell or CCR7^−^CD45RA^+^CD8^+^ T cell found in the regional lymph nodes of OSCC patients were dominated by the CD127^hi^ subsets (Fig. 2D). Representative data of FACS analysis of CD127 on CD8^+^ T cells from PBMC, tumor infiltrated cells or LN was shown in Figure S3 in File S1. These results indicated that CD127^ lo^CCR7^−^CD45RA^−^CD8^+^ T cell and CD127^lo^CCR7^−^CD45RA^+^CD8^+^ T cell are specific subsets of CD8^+^ T cells with increasing distribution frequency in circulation as well as tumor infiltrating lymphocytes of OSCC.

### CD127^hi^ and CD127^lo^CCR7^−^CD45RA^−^CD8^+^ or CCR7^−^CD45RA^+^CD8^+^ T cell are heterogeneous in surface molecule expression

Consistent with previous studies [11], the expression of CD28 correlated positively with that of CD127, with higher CD28^+^ distribution frequencies in the CD127^hi^ subset than that in the CD127^lo^ subset of either CCR7^−^CD45RA^−^CD8^+^ or CCR7^−^CD45RA^+^CD8^+^ T cell (Fig. S4 in File S1). More than 85% of the CD127^hi^ CCR7^−^CD45RA^−^CD8^+^ T cells were CD28^+^ and this correlation was consistently seen in CD8^+^ T cells isolated from normal donors or oral cancer patients (Fig. S4B, upper panel in File S1). About 60% of CD127^hi^ CCR7^−^CD45RA^+^CD8^+^ T cell cells expressed CD28, whereas only 20% of the CD127^lo^ CCR7^−^CD45RA^+^CD8^+^ T cell subset was CD28^+^, and similar distribution profiles were found in normal donors and oral cancer patients. No differences were found for PD-1 expression between CD127^hi^ or CD127^lo^ CCR7^−^CD45RA^−^CD8^+^ T cell and CCR7^−^CD45RA^+^CD8^+^ T cell subsets. Interestingly, approximately 50% of the CD127^lo^CCR7^−^CD45RA^−^CD8^+^ T cell or CCR7^−^CD45RA^+^CD8^+^ T cell subset was CD57^hi^, whereas less than 20% of CD127^hi^CCR7^−^CD45RA^−^CD8^+^ T cell or CCR7^−^CD45RA^+^CD8^+^ T cell subsets were CD57^+^ (Fig. S4B in File S1). These results indicated that CD127^hi^ or CD127^lo^CCR7^−^CD45RA^−^CD8^+^ T cell and CCR7^−^CD45RA^+^CD8^+^ T cell subsets are heterogeneous in terms of their surface expression of costimulatory molecules.

### CD127^hi^ or CD127^lo^ CCR7^−^CD45RA^−^CD8^+^ T cell and CCR7^−^CD45RA^+^CD8^+^ T cell exhibit consistent functional characteristics for IFN-γ and IL-2 production

**Figure 3:**
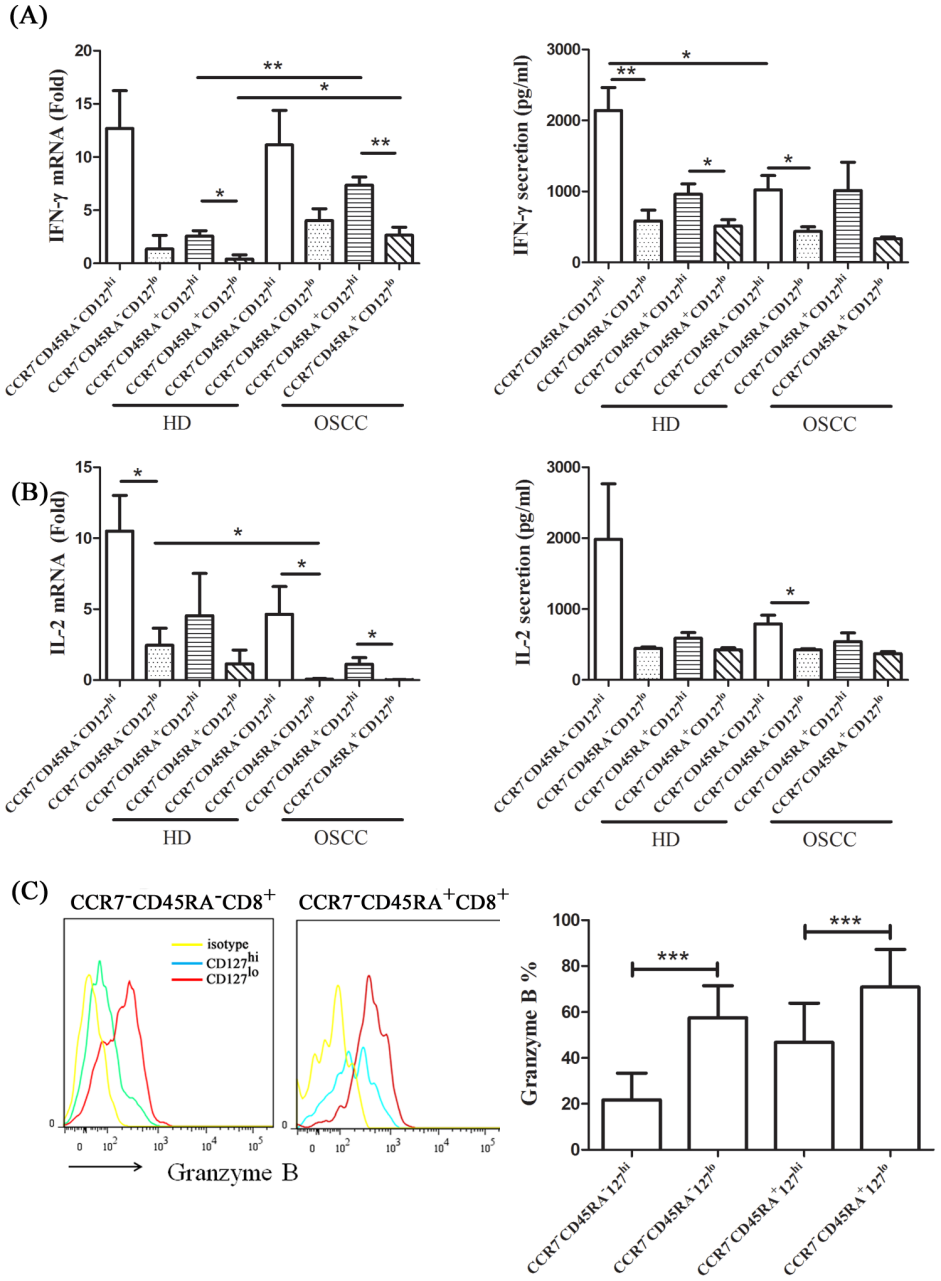
Different cytokine productivity of CD127^hi^ or CD127^lo^ CCR7^−^CD45RA^−^CD8^+^ T cell and CCR7^−^CD45RA^+^CD8^+^ T cell subsets. CD127^hi or lo^ cells in CCR7^−^CD45RA^−^CD8^+^ T cell and CD127^hi or lo^ cells in CCR7^−^CD45RA^+^CD8^+^ T cell were sorted from healthy donor, n = 5 or OSCC patients, n = 5. Four defined groups of CD8^+^ T cells were stimulated with anti-CD3 plus anti-CD28 coated beads (beads: cells = 1: 2) for 6 hours. Messaged RNA expression and secretion of (A) IFN-γ and (B) IL-2 were analyzed by qPCR and flowcytomix. The data were presented as the mean of related fold to GAPDH and mean (pg/ml) ± SD, respectively. Granzyme B expression in peripheral CD127^hi or lo^ CCR7^−^CD45RA^−^CD8^+^ T cell or CCR7^−^CD45RA^+^CD8^+^ T cell cells from OSCC patients (n = 12) was detected by intracellular staining (C). Yellow, isotype control; blue, CD127^hi^ CCR7^−^CD45RA^−^CD8^+^ T cell or CCR7^−^CD45RA^+^CD8^+^ T cell; red, CD127^ lo^ CCR7^−^CD45RA^−^CD8^+^ T cell or CCR7^−^CD45RA^+^CD8^+^ T cell. Significant differences compared with each group using *t*-tests are indicated by asterisks (*p<0.05, **p<0.01 and ***p<0.001).

To examine potential functional differences in cytokine production between CD127^hi^ and CD127^lo^ memory CD8^+^ T cells, we investigated the ability of these cells to produce IL-2 and IFN-γ following cell-sorting and *in vitro* stimulation with anti-CD3/anti-CD28-coupled Dynabeads [27]. Most interestingly, CD127^hi^ CCR7^−^CD45RA^−^CD8^+^ T cell and CCR7^−^CD45RA^+^CD8^+^ T cell cells expressed and secreted significantly higher amounts of IL-2 than CD127^lo^ counterparts (Fig. 3A & B). The CD127^lo^ CCR7^−^CD45RA^−^CD8^+^ T cell or CCR7^−^CD45RA^+^CD8^+^ T cell subsets exhibited negligible expression of IL-2 mRNA, whereas the average expression level of IL-2 mRNA was 100- fold higher in the OSCC groups, and 4- fold higher in the HD group in CD127^hi^ CCR7^−^CD45RA^−^CD8^+^ T cell or CCR7^−^CD45RA^+^CD8^+^ T cell subsets upon anti-CD3 and anti-CD28 stimulation. Moreover, the CD127^lo^CCR7^−^CD45RA^−^CD8^+^ T cell or CCR7^−^CD45RA^+^CD8^+^ T cell subsets exhibited a significantly higher activity in either absolute granzyme B producing levels or relative frequencies of granzyme B-expressing cells occur in oral cancer patients (Fig. 3C).

### CD127^hi^ or CD127^lo^ CCR7^−^CD45RA^−^CD8^+^ T cell and CCR7^−^CD45RA^+^CD8^+^ T cell exhibit consistent ex vivo proliferative capacity

**Figure 4:**
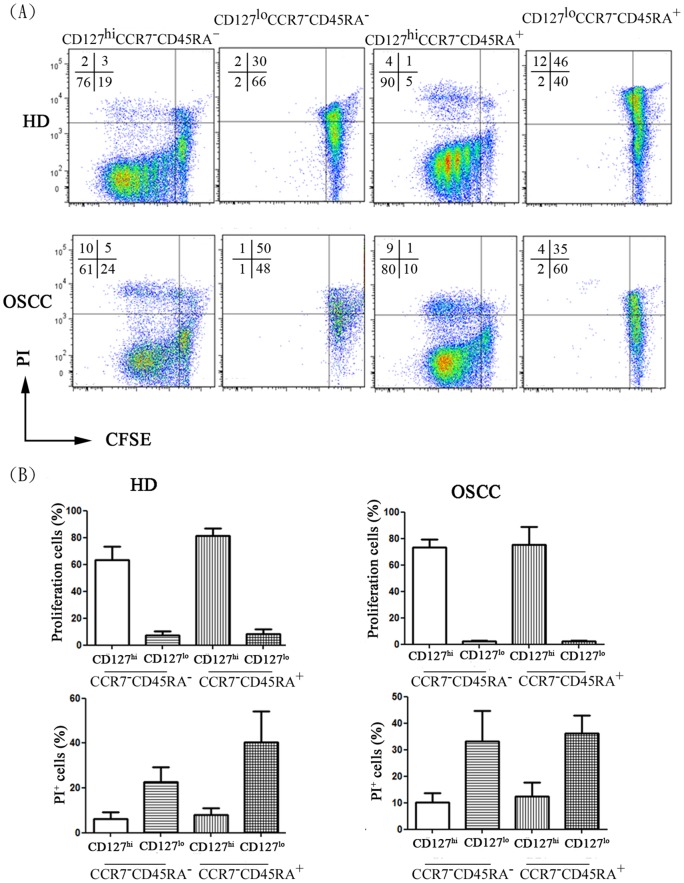
The proliferation and cell death of activated CD127^hi^ or CD127^lo^ CCR7^−^CD45RA^−^CD8^+^ T cell and CCR7^−^CD45RA^+^CD8^+^ T cell subsets. (A) Proliferation and cell death of CD8^+^ T subsets was analyzed by flow cytometry. Four defined groups were sorted and labeled with CFSE and were stimulated with anti-CD3 plus anti-CD28 coated beads. After 5 days, the cells were stained with propidium iodide (PI) to define dead cells. The percentage of proliferating and dying cells from different individuals (healthy donor, n = 3; OSCC patients, n = 3) are depicted in (B).

Judging from the IL-2 productivity of the CD127^hi^ or CD127^lo^ CCR7^−^CD45RA^−^CD8^+^ T cell andCCR7^−^CD45RA^+^CD8^+^ T cell subsets, we hypothesized that the CD127^lo^ subset, not the CD127^hi^ subset, represented terminally differentiated effector CD8^+^ T cells, thought to be non-replicative and more susceptible to activation-induced cell death [28]. Both CD127^hi^ CCR7^−^CD45RA^−^CD8^+^ T cell and CCR7^−^CD45RA^+^CD8^+^ T cell subsets, isolated from each group of 6 donors, proliferated significantly (over 60%) when they were cultured in medium without exogenous IL-2 (Fig. 4A & B). These results indicate that activated CD127^hi^ CCR7^−^CD45RA^−^CD8^+^ or CCR7^−^CD45RA^+^ CD8^+^ cells can proliferate regardless of exogenous IL-2, whereas activated CD127^lo^CCR7^−^CD45RA^−^CD8^+^ or CCR7^−^CD45RA^+^CD8^+^ T cell resembled terminally differentiated effector cell phenotypes characterized by the lack of IL-2 productivity and *ex vivo* proliferative activity.

In parallel experiments, we analyzed the susceptibility of these CD127^hi^ or CD127^lo^ subsets to succumb to activation-induced cell death after TCR stimulation. In all tested groups of donors, the percentage of PI^+^ cells was significantly higher in the CD127^lo^ CCR7^−^CD45RA^−^CD8^+^ or CCR7^−^CD45RA^+^CD8^+^ T cell subset (23 to 33% and 33 to 40%, respectively) than in their CD127^hi^ counterparts (6 to 11% for CCR7^−^CD45RA^−^CD8^+^ T cell and 8 to 17% for CCR7^−^CD45RA^+^CD8^+^ T cell) (Fig. 4). These findings indicate that CD127^hi^ CCR7^−^CD45RA^−^CD8^+^ T cell or CCR7^−^CD45RA^+^CD8^+^ T cell cells are more resistant to activation induced cell death than are CD127^lo^ cells. Together, these results suggested that CD127^ lo^ CCR7^−^CD45RA^−^CD8^+^ T cell or CCR7^−^CD45RA^+^CD8^+^ T cell maintain functional characteristics as terminally differentiated effectors [28] while CD127^hi^ CCR7^−^CD45RA^−^CD8^+^ T cell or CCR7^−^CD45RA^+^CD8^+^ T cell subsets that have consistently higher proliferative capacity and IL-2 productivity preserve memory phenotypes.

### Detection of antigen-specific CCR7^−^CD45RA^−^CD8^+^ T cell and CCR7^−^CD45RA^+^CD8^+^ T cell in OSCC patients

**Figure 5:**
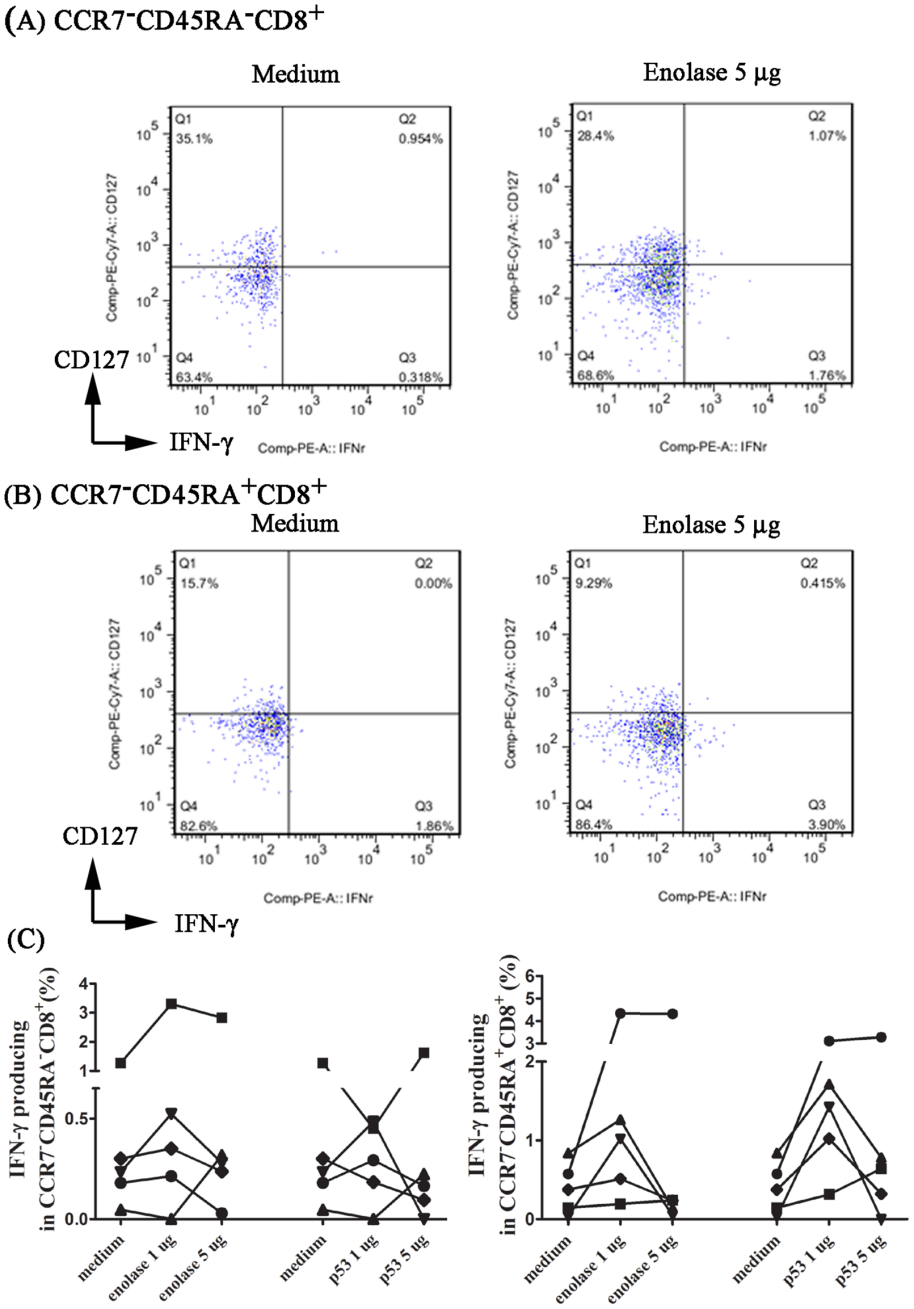
Antigen-specific recall responses of CD8^+^ T cells from OSCC patients. PBMC from 5 OSCC patients were cultured with or without alpha-enolase peptide-mixture or p53 peptide. The frequency (%) of IFN-γ producing cells in the total CCR7^−^CD45RA^−^CD8^+^ T cell or CCR7^−^CD45RA^+^CD8^+^ T cell population of each subset is detected by flow cytometry after intra-cellular staining. Representative result from one donor was depicted (A) CCR7^−^CD45RA^−^CD8^+^ T cell and (B) CCR7^−^CD45RA^+^CD8^+^ T cell. Data of frequency (%) of IFN-γ producing cells in CCR7^−^CD45RA^+^CD8^+^ T cell or CCR7^−^CD45RA^−^CD8^+^ T cell from five individuals and nonspecific cytokine-positive cells (cytokine-positive cells in the coculture without peptide) were marked as medium were depicted (C).

In western countries, several up-regulated tumor associated antigens of OSCC including alpha-enolase, heat shock proteins, p53 and their related auto-antibodies have been identified through genetic or proteomic approaches [29]–[30]. Antigen-specific CD8^+^ T-cell response has also been demonstrated using peptide-mix derived from alpha enolase or p53_264–272_ peptide in patients with pancreatic or head and neck cancer in Northern American. Therefore, we examined if α-enolase and p53 may serve as potential antigens to induce specific CD8^+^ effector memory repose in OSCC patients. The PBMC from OSCC patients was cultured in vitro in the presence or absence of a peptide scrambles containing 13 peptides, or p53_264–272_ predicted to bind to HLA –A*0201, which is the predominant MHC class I allele in around of 4% of Taiwanese [31]–[32]. Among 5 OSCC (HLA –A*02 haplotype) patients tested, two of them exhibited dose-dependent IFN-γ producing CCR7^−^CD45RA^−^CD8^+^ T cell, another volunteer showed dose-dependent response in CCR7^−^CD45RA^+^CD8^+^ T cell induced by enolase-peptides. Only one patient (HLA –A*0201 haplotype) exhibited dose-dependent response of CCR7^−^CD45RA^+^CD8^+^ T cell induced by p53 peptide (Fig. 5). However, CCR7^−^CD45RA^−^CD8^+^ T cell or CCR7^−^CD45RA^+^CD8^+^ T cell antigen-specific recall responses in these patients are considerably low (Fig. 5). In addition, the tetrameric staining of p53_264–272_ in CD8^+^ tumor infiltrating lymphocytes was not performed and the HLA –A*02 haplotype responsive epitope derived from α-enolase was not identified in the present study. Therefore, whether p53 or α-enolase is tumor associated antigen for OSCC in Taiwanese or Chinese population awaits further investigation.

### Association of CCR7^−^CD45RA^−^CD8^+^ T cell or CCR7^−^CD45RA^+^CD8^+^ T cell with tumor progression in OSCC

The presence of functionally distinct CCR7^−^CD45RA^−^CD8^+^ or CCR7^−^ CD45RA^+^CD8^+^ T cell subsets leads us to investigate the association of their distribution frequency with the histopathological staging of OSCC patients. Statistically significant correlation was found between the distribution frequency of CCR7^−^CD45RA^−^CD8^+^ T cell or CCR7^−^CD45RA^+^CD8^+^ T cell subset and the tumor staging, with CCR7^−^CD45RA^+^CD8^+^ T cell correlated with more advanced staging while the CCR7^−^CD45RA^−^CD8^+^ T cell with more earlier staging. Coincidentally, we also found that the ratio of CCR7^−^CD45RA^+^CD8^+^ T cell to CCR7^−^CD45RA^−^CD8^+^ T cell was also significantly correlated (*p*<0.05) with the advanced staging and with the poor differentiation status of the tumor cells in the OSCC patients enrolled in the current study (Table 1). The plasmatic concentration of the IL-7 was measured by ELISA in OSCC patients compared with healthy controls. We observed that the mean concentration of plasmatic IL-7 was significantly higher in the OSCC patients than that found in healthy donors; 81.90±5.645 versus 50.76±7.197 pg/ml (Fig. S5 in File S1). Therefore, elevated plasmatic IL-7 may sustain the increased distribution of CD127^lo^ CCR7^−^CD45RA^−^CD8^+^ T cell and CCR7^−^CD45RA^+^CD8^+^ T cell subsets in the circulation of cancer patients or may be due to that CCR7^−^CD45RA^+^CD8^+^ T cell subsets consumed less IL-7. In conclusion, our data suggested that dynamic changes between CCR7^−^CD45RA^−^CD8^+^ T cell or CCR7^−^CD45RA^+^CD8^+^ T cell is found in the systemic immunity and shift toward CCR7^−^CD45RA^+^CD8^+^ T cell is associated with tumor progression in the OSCC patients.

## Discussion

A most recent clinical trial indicated that stoichiometric production of IFN-γ and IL-2 defines memory CD8^+^ T cells that can self-renew after adoptive transfer in humans [33]. We demonstrated in the present study that CD127 (IL-7Rα) can be easily adopted in routine clinical settings to enrich among the CD8^+^ populations CD127^hi^ CCR7^−^CD45RA^−^ and CCR7^−^CD45RA^+^, that maintain *ex vivo* proliferative capacity and IL-2 production. The distinct subset of CD127^lo^CCR7^−^CD45RA^+^CD8^+^ T cell in human circulation has been previously demonstrated to be largely late differentiated and replicative senescent [12]–[16]. We also demonstrated in the present report that CD127^lo^CCR7^−^CD45RA^+^CD8^+^ T cell not only exhibits low replicative capacity *in vitro* but also preserve a higher capacity to produce granzyme B but devoid of IL-2, sharing functional characteristics of terminally differentiated effectors of CD8^+^ T cells [34]–[35]. Furthermore, we provide novel information to indicate that in the CCR7^−^CD45RA^−^CD8^+^ T cell population of there is also CD127^lo^ subset which shares identical phenotypic and functional characteristics as CD127^lo^CCR7^−^CD45RA^+^CD8^+^ T cell. Therefore, CD127^lo^CCR7^−^CD45RA^−^CD8^+^ T cell also belongs to the late differentiated effector rather than an effector memory subset of human CD8^+^ T cells. The selective accumulation of both CD127^lo^ CCR7^−^CD45RA^−^CD8^+^ T cell and CCR7^−^CD45RA^+^CD8^+^ T cell during OSCC progression may suggest that continuous antigenic stimuli or exposure were present to foster the peripheral homeostasis of CD8^+^ T cell pools skewing toward fully differentiated effector lymphocytes. The increase distribution frequency of both CD127^lo^ CCR7^−^CD45RA^+^CD8^+^ T cell and CCR7^−^CD45RA^−^CD8^+^ T cell in the circulation of OSCC patients accompanied with their increased susceptibility to activation induced cell death may account for the increased susceptibility of circulating CD8^+^ T cells to apoptosis observed earlier by others [15]–[16].

It is interesting to note that OSCC progressed despite of an increased CD8^+^ effector memory response in the circulation as well as in the tumor infiltrating lymphocytes. It has been proposed that OSCC exhibits an immunosuppressive microenvironment [7]–[8] and tumor cells could exhibit proficient capacity to express Fas ligand to induce apoptosis of T cells [16] or to produce indoleamine 2,3-dioxygenase (IDO) to inhibit the local proliferation of effector T cells and induce the regulatory T cells [36]–[37]. We have recently demonstrated that, in OSCC, tumor nests are infiltrated by distinct subsets of CD4^+^FOXP3^+^ regulatory T cells [23]. It is also found that in patients with oral cancer the circulating or tumor infiltrating CD8^+^ T cells are more susceptible to apoptosis than the CD4^+^ T cells [16]. We provide novel information to indicate that among both CCR7^−^CD45RA^–^CD8^+^ and CCR7^–^CD45RA^+^CD8^+^ T cells there are two functionally distinct populations exhibiting differential susceptibility to activation induced cell death. Therefore, it is possible that systemic anti-tumor response exerted through CD8^+^ effector memory cells may still exist despite of presence of local immunosuppressive microenvironment notably found in OSCC.

The most interesting finding of the present study is that the dynamic change of the circulating effector memory CD8^+^ T cell pools occurred during tumor progression with the distribution frequency of CCR7^–^CD45RA^–^CD8^+^ T cell and CCR7^–^CD45RA^+^CD8^+^ T cell negatively correlated with each other. In addition, the balance between these two subsets is associated with the tumor staging. The fact that CCR7^–^CD45RA^–^CD8^+^ T cell or CCR7^–^CD45RA^+^CD8^+^ T cell in the regional lymph nodes outnumbered by the CD127^hi^ subsets may suggest that the CD127^hi^CCR7^–^CD45RA^–^CD8^+^ T cell or CCR7^–^CD45RA^+^CD8^+^ T cell, which exhibit functionally characteristics as self-renewing effector memory cells, could be expanded in the regional lymph nodes during OSCC progression. In contrast, the CD127^lo^CCR7^–^CD45RA^–^CD8^+^ T cell or CCR7^–^CD45RA^+^CD8^+^ T cell, resembling the terminally differentiated effectors outnumbered in the tumor infiltrating lymphocytes or circulating CD8^+^ pools.

The mechanisms that control homeostatic proliferation of peripheral T-cell populations largely remain to be elucidated. In mouse, IL-7 plays an important role in the homeostasis and survival of peripheral CD8^+^ T cells through the regulation of IL-7 receptor-α expression and up-regulating the expression of anti-apoptotic Bcl-2 family proteins [38]–[39]. The increased plasmatic IL-7 levels and increased distribution of CCR7^−^CD45RA^−^CD8^+^ T cell or CCR7^−^CD45RA^+^CD8^+^ T cell subsets demonstrated in the present study, suggesting that OSCC patients may not be more immunocompromised than the healthy donors. Nevertheless, the skewing of peripheral CD8^+^ T-cell pools away from the naive subsets as demonstrated in the OSCC patients may suggest that chronic exposure to tumor-associated antigens, like p53 or enolase, tends to drive the naïve cells to effector memory and to terminally differentiated effectors. A most recent study demonstrated that such altered pattern of CD8^+^ T-cell differentiation in head and neck cancer patients could drive the circulating CD8^+^ peripheral memory (T_PM_) and CCR7^−^CD45RA^−^CD8^+^ T cell toward a higher rate of apoptosis than in healthy controls [14]. Moreover, the shortage of circulating naive T-cell pool as found in elderly people (more 80 years old) may account for the higher susceptibility to severe infectious diseases [40]. Therefore, the skewing of circulating CD8^+^ T cell pools toward CCR7^−^CD45RA^+^CD8^+^ T cell along with an elevated IL-7 level may represent another aspect of tumor derived immunosubversion mechanisms in human.

## Supporting Information

File S1 Supporting figures and tables. **Figure S1**, the correlation of CD127^hi^ or CD127 ^lo^ CCR7^−^CD45RA^+/−^CD8^+^ T cells with age. Healthy donor (A); OSCC patient (B). Significant differences compared with each group using Spearman correlation tests are indicated by asterisks (*p<0.05, **p<0.01). **Figure S2**, the distribution of CD127^hi^ or CD127 ^lo^ CCR7^−^CD45RA^+/−^ CD8^+^ T cell between age-matched healthy donors and OSCC patients. The expression of (A) CD127^hi^ or (B) CD127^lo^ in CCR7^−^CD45RA^−^CD8^+^ T cell; or (C) CD127^hi^ or (D) CD127^lo^ in CCR7^−^CD45RA^+^CD8^+^ T cell were examined in PBMC from healthy donors (n = 11) or OSCC patients (n = 10) with matched age of 19 to 45 years old. Significant differences compared with each group using *t*-tests are indicated by asterisks (**p<0.01 and ***p<0.001). **Figure S3**, analysis of CD127 expressing CD8^+^ T cells by FACS. Representative FACS analysis of CD127 expression in CCR7^−^CD45RA^+/−^CD8+ T cells in healthy donor PBMC (A), or OSCC patient's PBMC (B), tumor infiltrated lymphocytes (C), and lymph node (D). **Figure S4,** characterization of the surface molecule expression in CD127^hi^ and CD127^lo^ CCR7^−^CD45RA^−^ or CCR7^−^CD45RA^−^ subsets. (A) The surface CD28, PD-1 and CD57 expression pattern on these four subsets of CD8^+^ T cells. The CD127^hi^ cells are presented as a solid line, and the CD127^lo^ cells are presented as a dotted line. (B) The columns indicate the percentage of surface marker expression in different individuals from three groups (Healthy donor, n = 3; OSCC patients, n = 6.). Significant differences compared with each group using t-tests are indicated by asterisks (*p<0.05, **p<0.01 and ***p<0.001). **Figure S5,** concentration of plasmatic IL-7 in OSCC and healthy donors. The IL-7 concentration was determined by ELISA. The data was represented as mean pg/ml ± SD (healthy donor, n = 28; OSCC patients, n = 64). Statistical analysis was done by t-tests (**p<0.01). **Table S1,** clinicopathologic characteristics of the patients with OSCC and normal controls.
(DOCX)
