# Supplementary material for: Skewed Distribution of IL-7 Receptor-α-Expressing Effector Memory CD8+ T Cells with Distinct Functional Characteristics in Oral Squamous Cell Carcinoma
Source: PLoS One. 2014 Jan 23;9(1):e85521. doi: 10.1371/journal.pone.0085521 (PMC3900423; doi:10.1371/journal.pone.0085521)

Table S1 Clinicopathologic characteristics of the patients with OSCC and normal controls

| Characteristics | OSCC (n=59)  N (mean) | Healthy donor (n=18)  N (mean) |  |
| --- | --- | --- | --- |
| **Age (range)** | 35-91 (56) | 19-61 (34) |  |
| **Gender** | | | |
| Male | 55 | 10 |  |
| Female | 4 | 8 |  |
| **Primary T stage** | | | |
| 1 | 11 |  |  |
| 2 | 17 |  |  |
| 3 | 11 |  |  |
| 4 | 17 |  |  |
| unknown | 3 |  |  |
| **Primary N stage** | | | |
| 0 | 36 |  |  |
| 1 | 13 |  |  |
| 2 | 7 |  |  |
| 3 | 1 |  |  |
| unknown | 2 |  |  |
| **Primary tumor site** | | | |
| Buccal mucosa | 17 |  |  |
| Gingiva | 6 |  |  |
| Tongue | 17 |  |  |
| Floor of mouth | 1 |  |  |
| Oropharynx | 5 |  |  |
| Palate | 3 |  |  |
| Lip | 1 |  |  |
| Retromolar area | 1 |  |  |
| Multiple | 4 |  |  |
| Unknown | 4 |  |  |
| **Invasion** | | | |
| Lymphovascular invasion | 17 |  |  |
| Perineural invasion | 23 |  |  |

Figure S1


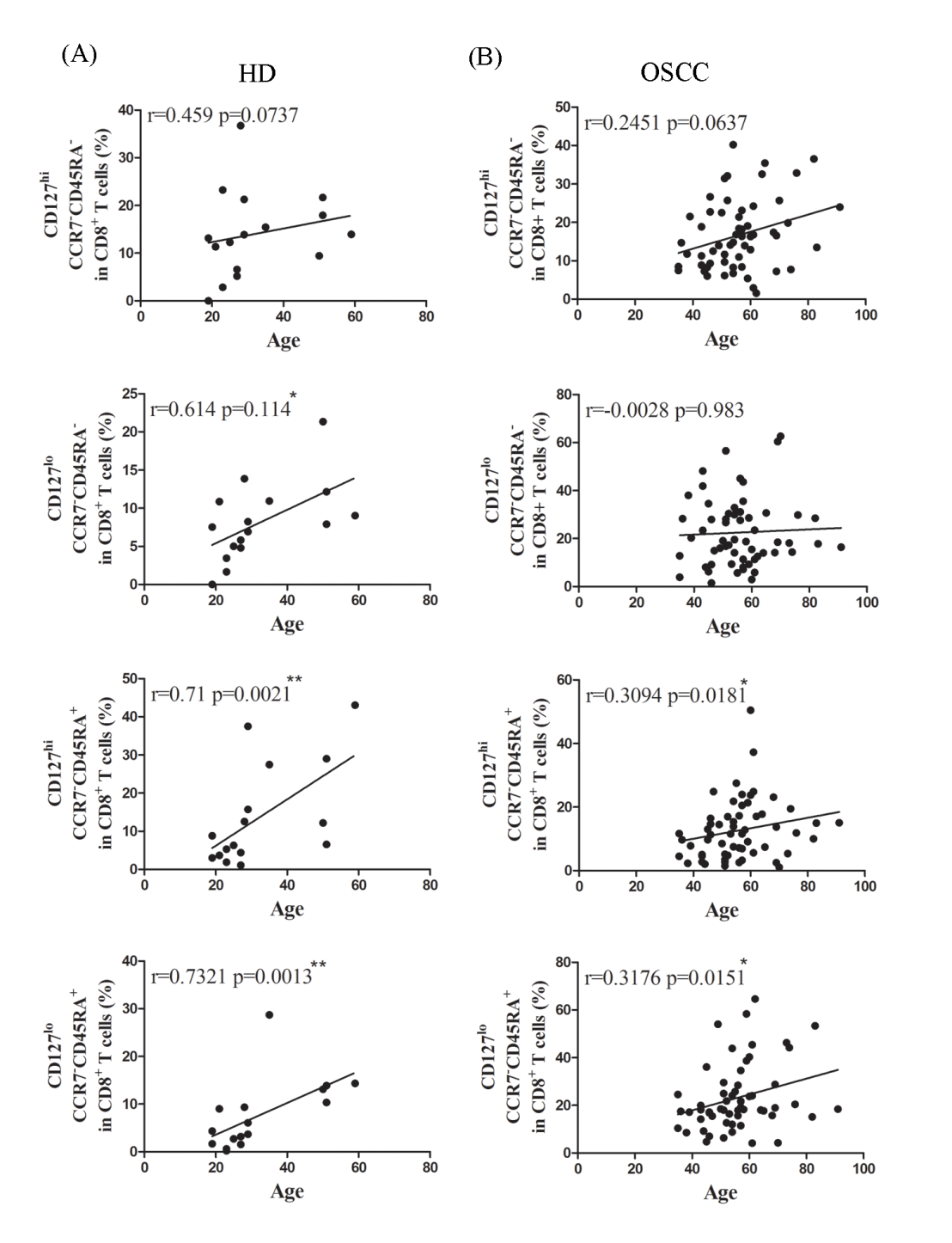


Figure S2


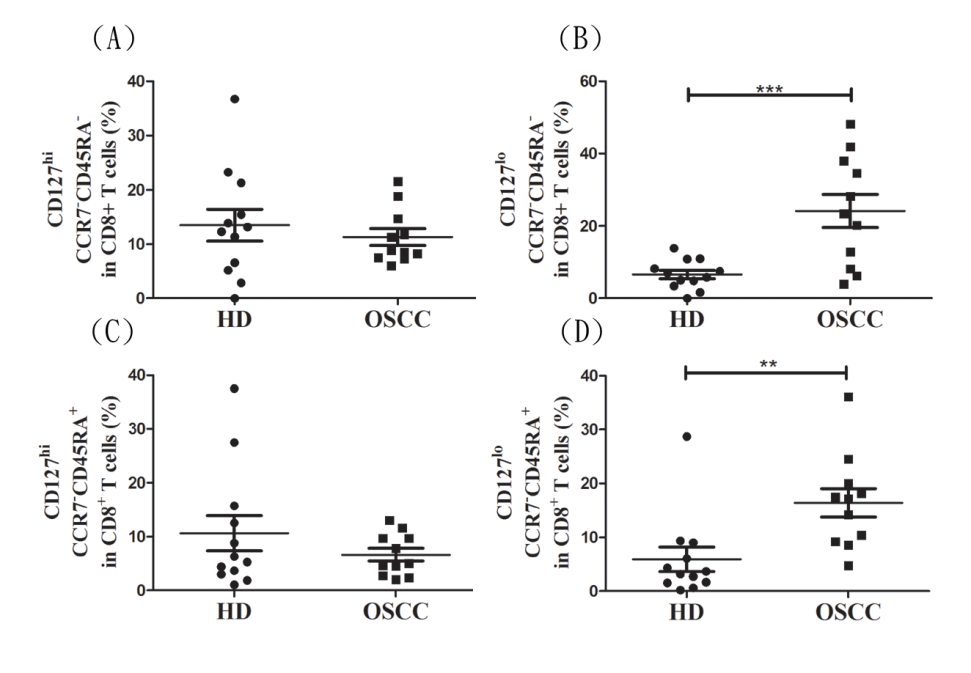


Figure S3


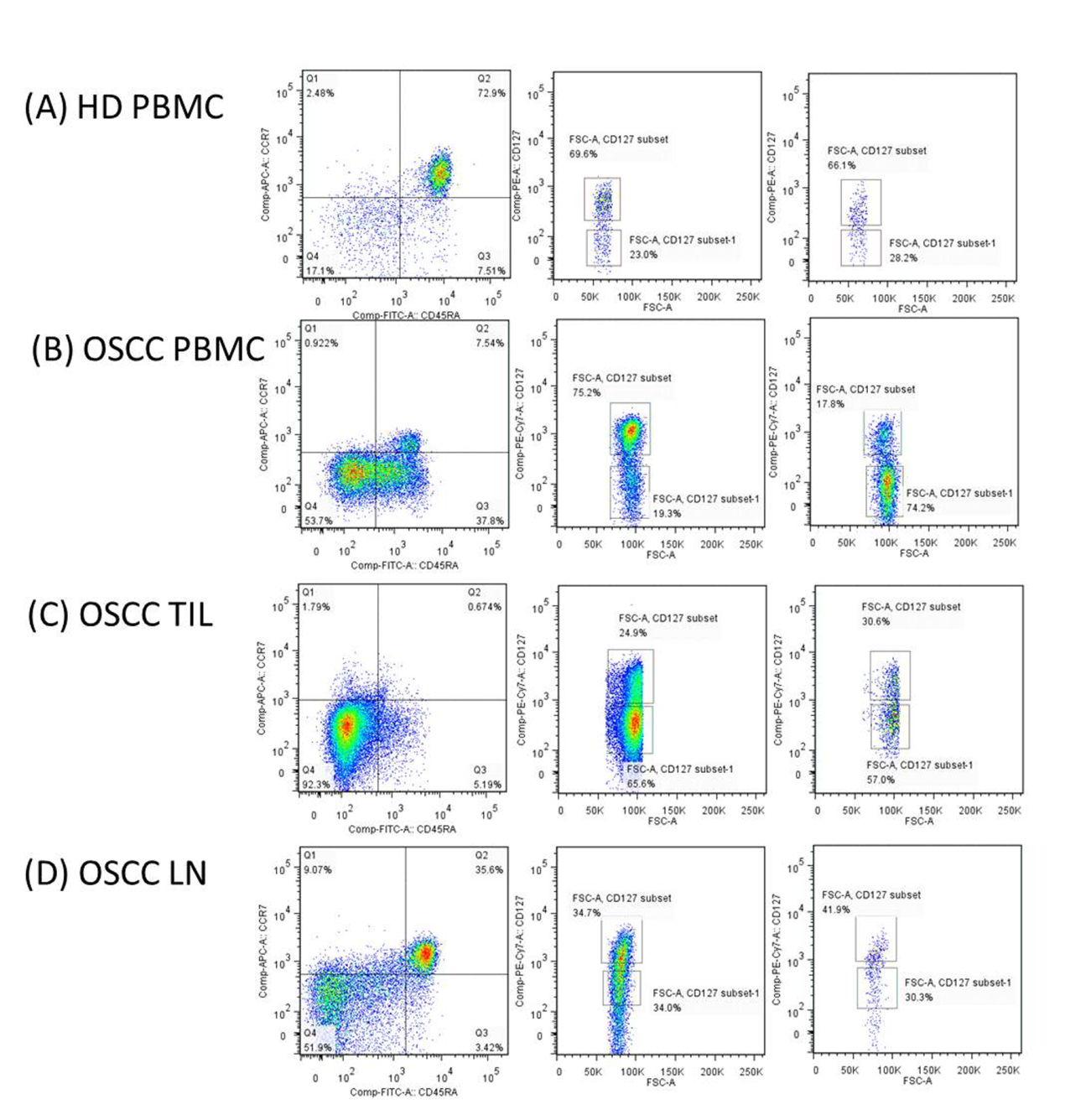


Figure S4


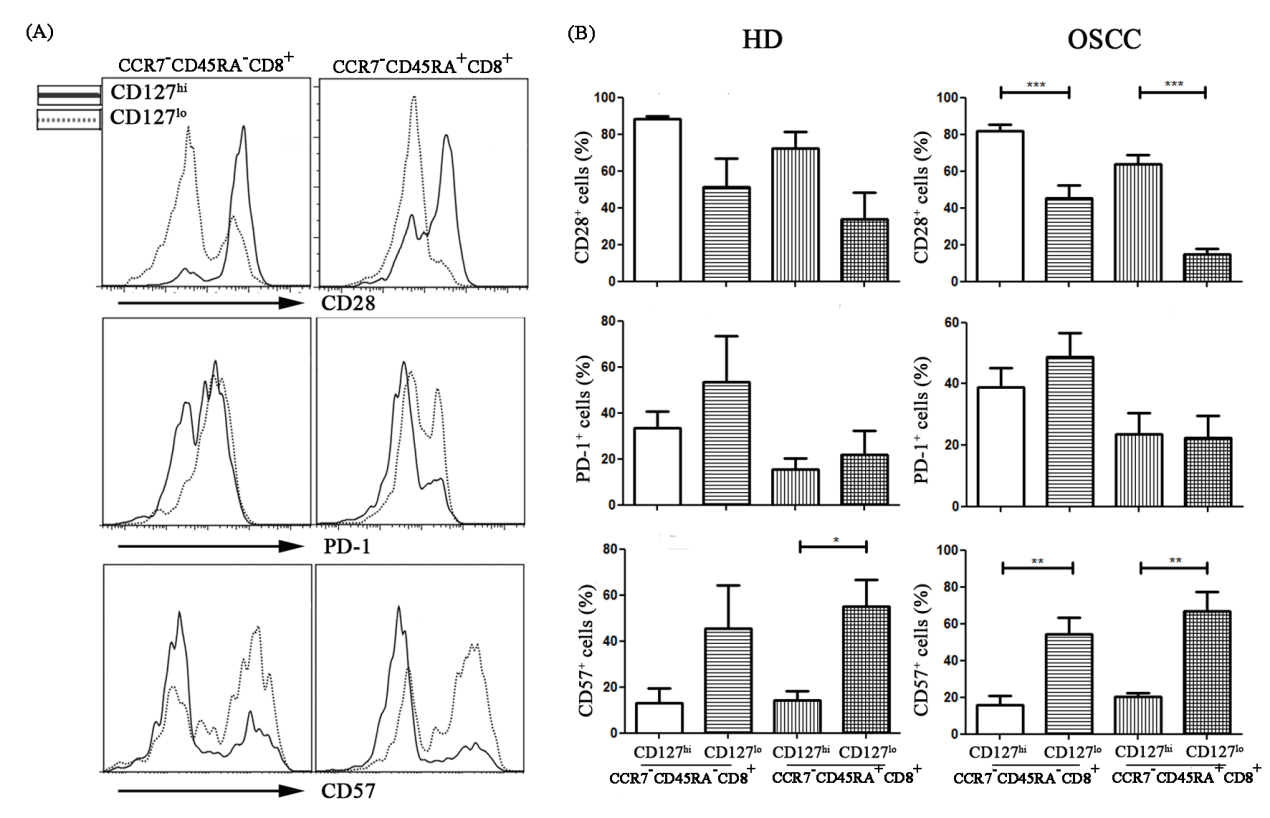


Figure S5


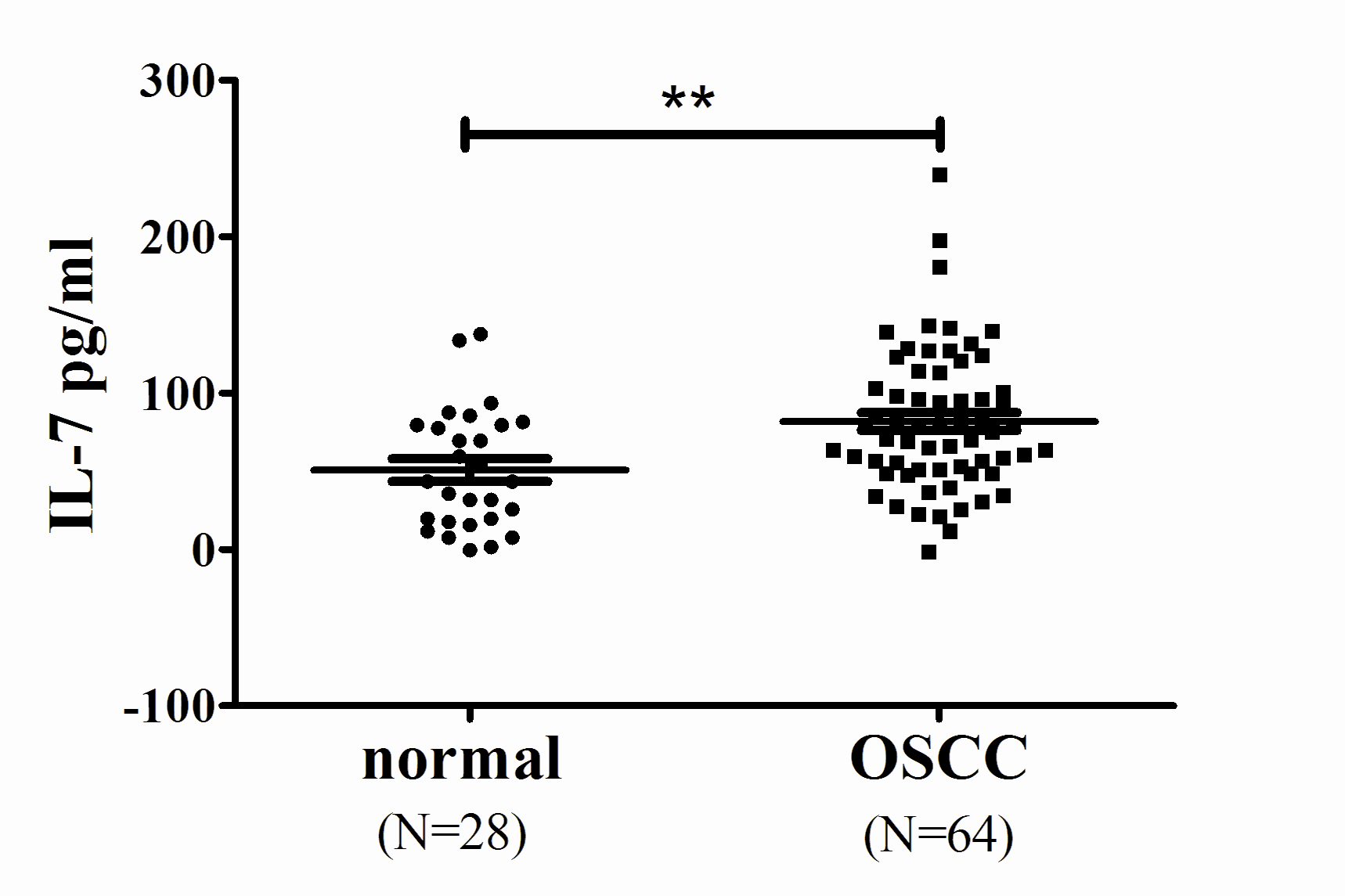

Supplement: File S1 — Supporting figures and tables. Figure S1, the correlation of CD127hi or CD127 lo CCR7−CD45RA+/−CD8+ T cells with age. Healthy donor (A); OSCC patient (B). Significant differences compared with each group using Spearman correlation tests are indicated by asterisks (*p<0.05, **p<0.01). Figure S2, the distribution of CD127hi or CD127 lo CCR7−CD45RA+/− CD8+ T cell between age-matched healthy donors and OSCC patients. The expression of (A) CD127hi or (B) CD127lo in CCR7−CD45RA−CD8+ T cell; or (C) CD127hi or (D) CD127lo in CCR7−CD45RA+CD8+ T cell were examined in PBMC from healthy donors (n = 11) or OSCC patients (n = 10) with matched age of 19 to 45 years old. Significant differences compared with each group using t-tests are indicated by asterisks (**p<0.01 and ***p<0.001). Figure S3, analysis of CD127 expressing CD8+ T cells by FACS. Representative FACS analysis of CD127 expression in CCR7−CD45RA+/−CD8+ T cells in healthy donor PBMC (A), or OSCC patient's PBMC (B), tumor infiltrated lymphocytes (C), and lymph node (D). Figure S4, characterization of the surface molecule expression in CD127hi and CD127lo CCR7−CD45RA− or CCR7−CD45RA− subsets. (A) The surface CD28, PD-1 and CD57 expression pattern on these four subsets of CD8+ T cells. The CD127hi cells are presented as a solid line, and the CD127lo cells are presented as a dotted line. (B) The columns indicate the percentage of surface marker expression in different individuals from three groups (Healthy donor, n = 3; OSCC patients, n = 6.). Significant differences compared with each group using t-tests are indicated by asterisks (*p<0.05, **p<0.01 and ***p<0.001). Figure S5, concentration of plasmatic IL-7 in OSCC and healthy donors. The IL-7 concentration was determined by ELISA. The data was represented as mean pg/ml ± SD (healthy donor, n = 28; OSCC patients, n = 64). Statistical analysis was done by t-tests (**p<0.01). Table S1, clinicopathologic characteristics of the patients with OSCC and normal controls. (DOCX) [file pone.0085521.s001.docx]
